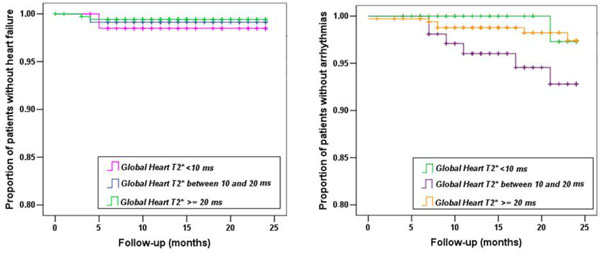# Heart T2* for prediction of cardiac complications in well-treated thalassemia major patients

**DOI:** 10.1186/1532-429X-14-S1-P195

**Published:** 2012-02-01

**Authors:** Alessia Pepe, Antonella Meloni, Giuseppe Rossi, Petra Keilberg, Cristina Salvatori, Elisabetta Chiodi, Claudio Ascioti, Antonella Carollo, Vincenzo Positano, Massimo Lombardi

**Affiliations:** 1 CMR Unit, Fondazione G.Monasterio CNR-Regione Toscana and Institute of Clinical Physiology, Pisa, Italy; 2 Epidemiology and Biostatistics Unit, Institute of Clinical Physiology, CNR, Pisa, Italy; 3 Dipartimento di Radiologia, Ospedale “Sant’Anna”, Ferrara, Italy; 4 Struttura Complessa di Cardioradiologia-UTIC, P.O. “Giovanni Paolo II”, Lamezia Terme, Italy; 5 Servizio Talassemia-U.O. Pediatria Talassemia, Az. Osp. "Sant'Antonio abate", Trapani, Italy

## Background

T2* Magnetic Resonance Imaging (MRI) technique allows noninvasive quantification of organ-specific iron burden, playing a key role in the management of thalassemia major (TM) patients. There are few data on the incidence of heart failure and arrhythmias in TM patients according to baseline T2* values. So, the aim of this study was to establish prospectively the risk of cardiac complications in a large cohort of well-treated TM patients.

## Methods

We considered 527 TM patients (252 males, mean age 30±9) for who clinical data relative to a period of 5 years after the first MRI were collected in a central data base. At time of the first scan mean ferritin levels were1653±1559 ng/l, global heart was 27±13 ms, and excellent/good level of compliance were present in the 96% of the study population.

## Results

At 5 years of follow-up, we recorded 24 cardiac events: 4 episodes of cardiac failure, 15 of arrhythmia, 1 of pulmonary hypertension and 4 of other cardiac complications. The majority of these events (21/24) happened within the first 24 months subsequent to the MRI, so we considered this follow-up period.

At the first MRI scan, in patients with cardiac complications the global heart T2* was 22.5 ±12.4 ms. In comparison with global heart T2* values ≥20 ms, there was not a significantly increased risk of cardiac complications associated with global heart T2* values <20 ms (HR= 2.028 P=0.09).

In the heart failure patients the global heart T2* was 19±12 ms. In comparison with global heart T2* values ≥20 ms, there was not a significantly increased risk of heart failure associated with global heart T2* values <20 ms (HR=1.9 P=0.524) or <10 ms (HR=2.6 P=0.443).

In the arrhythmic patients the global heart T2* was 25±13 ms. In comparison with global heart T2* values ≥20 ms, there was not a significantly increased risk of arrhythmia associated with global heart T2* values <20 ms (HR=2.1 P=0.179) or <10 ms (HR=0.8 P=0.824).

During the follow up changes in the chelation therapy (type and/or dose-frequencies) were found in > 25% of the study population.

## Conclusions

We detected very few cardiac events, almost all concentrated in the first 24 months. In a large cohort of well-treated TM patients heart T2* lost its power in predicting cardiac events probably due to a patient-specific adjustment of the chelation therapy MRI-guided.

## Funding

“No-profit” support by industrial sponsorships (Chiesi, Apotex and GE Healtcare) and “Ministero della Salute, fondi ex art. 12 D.Lgs. 502/92 e s.m.i., ricerca sanitaria finalizzata anno 2006” e “Fondazione L. Giambrone”.

**Figure 1:**